# Optimizing Prognostic Assessment in High‐Risk Head and Neck Squamous Cell Carcinomas: The Impact of Tumor Budding and a Novel Histomorphological Scoring System

**DOI:** 10.1002/cam4.70685

**Published:** 2025-02-15

**Authors:** Moritz Knebel, Jan Philipp Kühn, Sandrina Körner, Felix Braun, Lukas Brust, Silke Wemmert, Sigrun Smola, Martin Ertz, Mathias Wagner, Bernhard Schick, Luc G. T. Morris, Maximilian Linxweiler, Gilbert Georg Klamminger

**Affiliations:** ^1^ Institute of Otorhinolaryngology Saarland University Homburg Germany; ^2^ Department of Otorhinolaryngology, Head and Neck Surgery Saarland University Medical Center (UKS) Homburg Germany; ^3^ Institute of Virology Saarland University Medical Center Homburg/Saar Germany; ^4^ Helmholtz Institute for Pharmaceutical Research Saarland (HIPS) Helmholtz Centre for Infection Research Saarbrücken Germany; ^5^ Department of General and Special Pathology Saarland University Medical Center (UKS) Homburg Germany; ^6^ Department of Surgery Memorial Sloan Kettering Cancer Center New York City New York USA; ^7^ Experimental Cancer Immunogenomics Laboratory, Department of Surgery Memorial Sloan Kettering Cancer Center New York City New York USA; ^8^ Department of Obstetrics and Gynecology University Medical Center of the Johannes Gutenberg University Mainz Mainz Germany

**Keywords:** HNSCC, prognostication, tumor budding

## Abstract

**Background:**

Head and neck squamous cell carcinomas (HNSCC) pose significant clinical challenges, particularly in high‐risk cases with positive lymph node status. Current prognostic biomarkers are often costly and methodologically demanding. In this regard, histomorphological biomarkers such as tumor buds (TB) and poorly differentiated clusters (PDC) represent promising, cost‐effective prognostic indicators that are relatively straightforward to implement.

**Methods:**

The prognostic significance of TB and PDC, in conjunction with stromal tumor‐infiltrating lymphocytes (sTILs) and the tumor‐stroma ratio (TSR), was evaluated in a cohort of 50 high‐risk, nodal‐positive HNSCC patients. Histomorphological features were assessed using standard hematoxylin and eosin (H&E) staining, while HPV association and PD‐L1 expression were determined by means of immunohistochemistry (IHC) and/or PCR. All variables collected were subsequently correlated with traditional histopathological and clinical parameters. Finally, a novel scoring system incorporating TB and PDC was developed, and its association with overall survival (OS) was analyzed.

**Results:**

TB and PDC both demonstrated a significant impact on patients' OS (TB Log‐rank test, *p* = 0.0499, PDC Log‐rank test, *p* = 0.0235). A novel scoring system based on these features had strong association with patients' OS (Log‐rank test, *p* = 0.0200) in contrast to the conventional and routinely performed grading system, which evaluates the degree of differentiation within neoplastic cells (Log‐rank test, *p* = 0.3325). PD‐L1 expression was not associated with TB and PDC formation. HPV‐negative status was associated with a higher number of tumor buds.

**Conclusion:**

This study reveals the potential prognostic value of TB and PDC in high‐risk HNSCC, which may offer a practical and cost‐effective alternative to traditional markers. Our proposed practicable and straightforward employable scoring system significantly correlates with OS, suggesting its potential benefit in clinical practice. These findings advocate for further validation to enhance prognostic accuracy and guide treatment strategies in HNSCC.

## 1 Introduction

Head and neck squamous cell carcinoma (HNSCC) is the seventh most common cancer type worldwide, with approximately 890,000 estimated new cases annually [1]. Even though a broad variety of scientific findings and literature gathered within the last two decades have improved our understanding of the complex etiology and varying genetic and environmental risk factors underlying HNSCC [2, 3], there remain significant clinical unmet needs. The majority of patients with HNSCC are first diagnosed at a locally advanced stage and to date still face poor overall survival (OS) rates despite intense multimodal therapy [4, 5]. Given the variation in patient outcomes based on tumor stage and biology, a diagnostic strategy focused on individual risk stratification—utilizing both clinical and histomorphological biomarkers—could be beneficial in optimizing precision therapy [4].

A number of clinically useful prognostic biomarkers such as blood‐based analysis of circulating tumor cells and neutrophil to lymphocyte ratios [6, 7, 8, 9], tissue‐based markers based on immunohistochemistry (IHC) [10, 11, 12, 13], or biomarkers on a genetic level (RNA and DNA biomarkers) have been examined so far [14, 15]. Most accessible and globally available are traditional histologic features that can be discerned on routine hematoxylin and eosin (H&E) staining, for example, the morphology of cancer cells at the invasive tumor front. That said, the role of tumor‐intrinsic features such as the presence of tumor cell buds (tumor budding, TB) and poorly differentiated tumor nests (poorly differentiated clusters, PDC), as well as microenvironmental features such as the distribution of leukocytes within a desmoplastic stroma reaction (stromal tumor‐infiltrating lymphocytes, sTILs) has been previously studied [16, 17, 18, 19]. Nevertheless, the use of these factors in the routine diagnosis of head and neck cancer (HNC) is not yet established, due to a lack of diagnostic standards and accepted scoring or cutoff parameters.

To address this evidence gap, we here analyzed histomorphological factors such as TB, PDC, sTILs, and their correlation with established risk factors such as HPV tumor status and PD‐L1 expression, as well as their prognostic relevance in terms of OS. These analyses were performed in a cohort of patients with HNSCC and high‐risk clinical features, including positive lymph node status. To enhance prognostication through histopathological features, we also propose a novel grading system incorporating TB and PDC.

## 2 Material and Methods

### 2.1 Patient Data

In order to determine sufficient sample size, a priori sample size determination was employed with a required minimum sample size of *n* = 44. Tissue samples from a cohort of 50 HNSCC patients were collected from 2011 to 2021. All patients were diagnosed and treated at the Saarland University Medical Center (Homburg, Germany), and identified as high‐risk based upon lymph node status [20, 21]. Prior to study participation, informed consent was obtained. The study was approved by the Ethics Committee of Saarland (study identification number 218‐10) and all data were handled in alignment with the Declaration of Helsinki [22].

Overall, 44 (88%) male and 6 (12%) female patients, with a mean age of 62.6 years, were included. The median follow‐up period for patients was 37.5 months. Within our cohort, 72% of tumors were HPV‐negative, and 28% were tested positive for HPV. Table 1 gives an overview of clinical patient details.

**TABLE 1:** Clinical data overview of all 50 patients included.

Total number of patients	50	
Sex	Male	44 (88%)
	Female	6 (12%)
Median age	62.6 years	
HPV‐ Status	Positive	14 (28%)
	Negative	36 (72%)
Localization	Oropharynx	30 (60%)
	Larynx	9 (18%)
	Hypopharynx	7 (14%)
	Oral cavity	4 (8%)
T stage	1	5 (10%)
	2	23 (46%)
	3	11 (22%)
	4	11 (22%)
N stage	1	12 (24%)
	2	36 (72%)
	3	2 (4%)
M stage	0	46 (92%)
	1	4 (8%)
UICC Stage	I	7 (14%)
	II	6 (12%)
	III	0 (0%)
	IVa	33 (66%)
	IVb	1 (2%)
	IVc	3 (6%)
Therapy	Surgery alone	3 (6%)
	Surgery + RT	15 (30%)
	Surgery + CRT	30 (60%)
	Surgery + adjuvant RT + Cetuximab	2 (4%)

*Note:* TNM staging was performed according to the *TNM Classification of Malignant Tumors, 8th Edition*.

Abbreviations: CRT, chemoradiotherapy; RT, radiotherapy.

### 2.2 IHC and HPV Detection

The respective FFPE (formalin‐fixed paraffin embedded) tumor blocks of included patients were cut (4 μm) employing the Leica RM 2235 rotation microtome (Leica Microsystems, Wetzlar, Germany) before they were mounted from the water bath (46°C) to adhesive slides (Matsunami TOMO) and dried overnight at 37°C. Regular H&E staining (standard protocol) of the first cut served as a morphological control. PD‐L1 immunohistochemical detection was performed using the Benchmark Ultra system (Ventana Medical Systems) employing a primary antibody specific for PD‐L1 (Dako/Agilent, Santa Clara, CA, USA; clone 22C3; dilution 1:25) for 32 min at 37°C. The primary antibody bonding was subsequently marked using the ultraView Universal Alkaline Phosphatase Red Detection (Roche, Basel, Switzerland) according to the manufacturer's description. Antigen retrieval was performed with CC1 buffer (Ventana) for 64 min (at 97°C). We controlled for potential technical and biological confounders employing negative controls (omission of the primary antibody) and in‐house established suitable positive controls within every staining round. PD‐L1 expression was analyzed employing the “tumor proportion score” (TPS) and the “combined positivity score” (CPS). According to standard practice, the TPS takes into account the number of membranously stained tumor cells in relation to all vital tumor cells; the CPS is calculated as the relation of membranously stained tumor cells plus membranously or cytoplasmatically stained mononuclear immune cells (macrophages, lymphocytes, dendritic cells) and all vital tumor cells. Hereby, a minimum of > 100 vital cells was required for scoring; all non‐neoplastic epithelial cells as well as areas of necrosis and other cell types were not considered [23].

For immunohistochemical detection of p16, the CINtec p16 histology kit (Roche Diagnostics) was used according to the manufacturer's instructions. In brief, heat‐induced epitope unmasking was performed upon deparaffinization in a rice cooker for 20 min using the supplied retrieval buffer. Incubation with the p16 antibody and the detection of staining signals were performed as recommended by the manufacturer. Every staining series included negative and positive controls. Tissue stainings were rated p16 positive in the case of “block‐type” positive staining (strong cytoplasmatic and nuclear expression of at least 20 adjacent cells). Individual and single positive cytoplasmic stainings, the so‐called “patchy‐staining pattern” were not considered. The analysis was independently conducted by three examiners, including one board‐certified pathologist; discrepancies were resolved by consensus reaching discussion.

Within our study, HPV positivity was defined as positive HPV‐DNA‐PCR and a positive p16 immunohistochemical staining, taking into account the worse prognosis in case of discordant p16/HPV testing (p16‐/HPV+ or p16+/HPV‐) [24, 25]. HPV‐DNA‐PCR was performed after DNA extraction from frozen tumor samples employing the QIAamp DNA Blood Mini Kit (Qiagen, Hilden, Germany). As described elsewhere [26], subsequent amplification was performed employing GP5+/6+ primers and the LightCycler 2.0 system (Roche Diagnostics, Mannheim, Germany); the respective products were identified using SYBR Green as well as gel electrophoresis. After denaturation at 95°C (15 min), 45 PCR cycles with a denaturation at 95°C (10 s), annealing at 45°C (5 s), and elongation at 72°C (18 s) were conducted. A positive control served as an included HPV16 control (Tm 79°C)/HPV18 positive control (Tm 82°C) but furthermore, the additional amplification of the Glyceraldehyde‐3‐phosphat‐dehydrogenase (GAPDH) gene [27].

### 2.3 Histomorphological Biomarker Evaluation

All biomarker cutoffs were prespecified in this analysis, based on prior literature. Tumor slides were collected and re‐screened for histomorphological parameters of interest. By convention, TB are defined as a cluster of < 5 cells infiltrating tumor at the invasive front; infiltrating cell clusters consisting of five or more cancer cells, surrounded by stroma, were categorized as PDCs. Adherent to most studies as well as the 2016 consensus conference of the International Tumor Budding Consensus Conference (ITBCC) group, TB and PDCs were evaluated on H&E slides; to do so, 10 different fields of the invasive tumor front were screened (objective ×10) and the number of TB and PDCs were counted individually for one hotspot area (objective ×20) [18, 28, 29, 30]. To differentiate between TB‐positive carcinomas and TB‐negative carcinomas, we here employed the value of ≥ 3 tumor buds/field as the cutoff value [30, 31, 32]; a distinction between the groups PDC‐positive carcinomas and PDC‐negative carcinomas was achieved by defining a cutoff value of ≥ 4 per field for PDC‐positive carcinomas.

**FIGURE 1:**
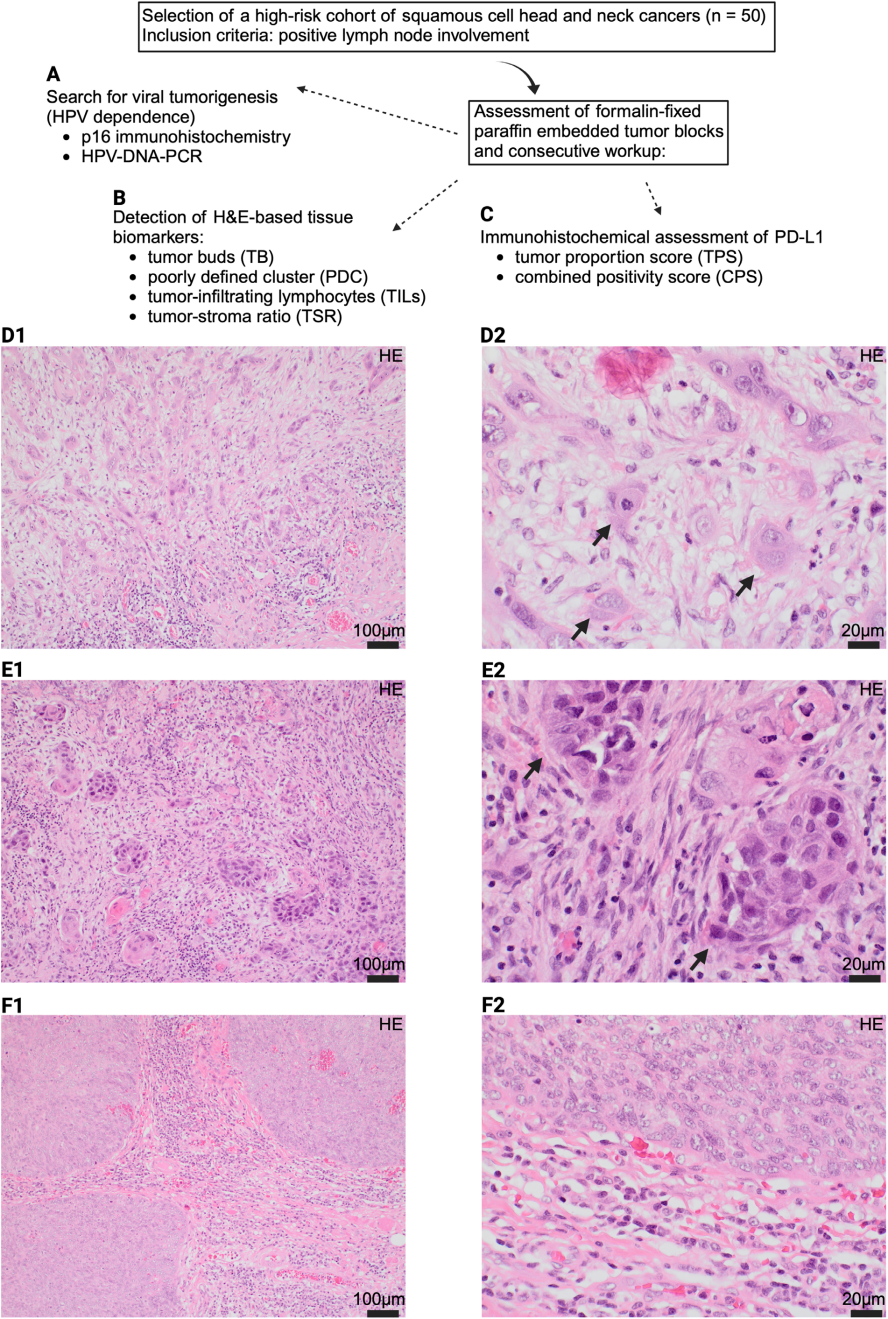
Visualization of our study protocol. After identification of a selected high‐risk patient cohort, tumor blocks were cut to subsequently analyze HPV status (p16 IHC and HPV‐DNA‐PCR; A), histomorphological biomarkers (B) and PD‐L1 status (C). D1 and D2 exhibit an infiltrating tumor area with prominent tumor bud formation (black arrows; tumor bud per definition < 5 neoplastic cells). E1 and E2 depict infiltrating tumor cell clusters with ≥ 5 cells (PDC). In F1 and F2 a tumor border without tumor bud formations and a moderate stromal lymphocytic infiltrate is presented.

As proposed by the International Immunooncology Biomarkers Working Group in a practical review, tumor‐infiltrating lymphocytes (TILs) of the stromal compartment at the invasive tumor margin were evaluated as a percentage of sTILs, defined by the stromal area occupied by mononuclear inflammatory cells (in %) at the invasive tumor border (namely an area of 1 mm at the invasive front, commonly within the desmoplastic stroma reaction) and three distinct groups were defined (based on the percentage of sTILs: group 1 0%–10%, group 2 11%–40%, group 3 41%–100%) [33]. Additionally, the relation of tumor cells to the stromal components within one area (objective ×10, where vital tumor components must be present on all sides of the light microscopical lens view) next to the invasive tumor front was rated as tumor‐stroma ratio (TSR) and classified as either stroma poor (less than 50% stromal components in comparison to vital tumor cells) or stroma rich (more than 50% stromal components in comparison to vital tumor cells) [34]. Figure 1 gives an overall insight into our study protocol.

### 2.4 Statistical Data Analysis

For statistical analyses, D'Agostino & Pearson omnibus normality test, Anderson‐Darling test, Shapiro–Wilk test, and Kolmogorov–Smirnov test were used to determine if datasets follow a Gaussian distribution in each comparison. Gaussian distribution was only assigned if the data sample passed ≥ 2 of the aforementioned normality tests. If the data showed a normal distribution, parametric tests were performed (two‐tailed unpaired *t*‐tests, one‐way ANOVA with Tukey's correction for multiple comparisons, or Pearson correlation). If the data showed no normal distribution, non‐parametric tests were applied (Mann–Whitney‐*U* test, one‐way ANOVA using Kruskal–Wallis with Dunn's correction for multiple comparisons, or Spearman correlation). For survival analyses comparing two groups, the log‐rank test based on the Mantel–Haenszel approach (Mantel‐Cox method) was employed. For analyses involving three or more groups, the same log‐rank test (Mantel‐Cox method) was utilized. Survival probabilities were reported either with standard errors or as 95% confidence intervals, with standard errors calculated using Greenwood's method. *p* values < 0.05 were considered statistically significant (*α* = 0.05).

## 3 Results

### 3.1 Prognostic Impact of TB, PDC, sTILs, and TSR

**FIGURE 2:**
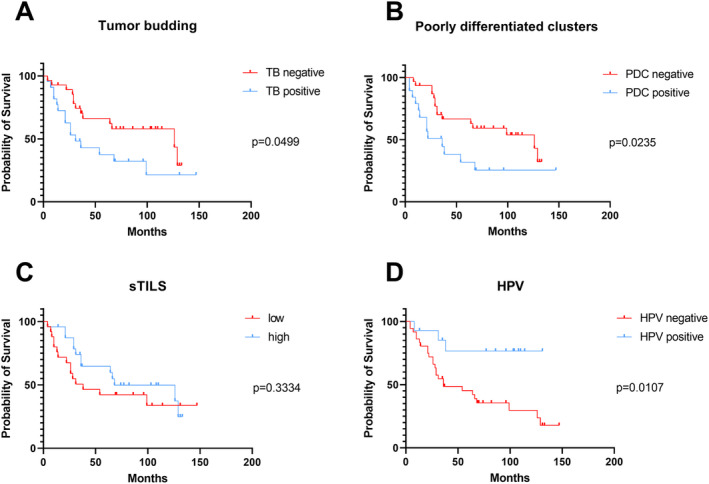
Prognostic relevance of TB, PDC, sTILS, and HPV tumor status within our cohort. (A) Patients' OS with TB‐positive and TB‐negative carcinomas, defined by a cutoff value of ≥ 3 buds/field. (B) Correlation of OS with PDC defined by a cutoff of ≥ 4 buds/field. (C) OS regarding high vs. low distribution of tumor‐infiltrating lymphocytes, defined by the median. (D) Patients' OS segregated by HPV tumor status. In A to D, a log‐rank test was used for statistical analysis.

To determine the prognostic value of these histological features, we examined their associations with OS. Our Kaplan–Meier analyses indicated the prognostic importance of TB (Log‐rank (Mantel‐Cox) test, *p* = 0.0499, HR = 0.4577, 95% CI = 0.2096 to 0.9998), PDC (Log‐rank (Mantel‐Cox) test, *p* = 0.0235, HR = 0.3772, 95% CI = 0.1623 to 0.8767) as well as HPV tumor status (Log‐rank (Mantel‐Cox) test, *p* = 0.0107, HR = 2.840 and 95% CI = 1.274 to 6.328). Specifically, reduced TB and PDC, along with a positive HPV tumor status, were associated with improved patient outcomes (see Figure 2). Conversely, we did not observe the prognostic importance of sTILs (Log‐rank (Mantel‐Cox) test, *p* = 0.7745, sTILS: HR = 1.450, 95% CI = 0.6829 to 3.080) and TSR (Log‐rank (Mantel‐Cox) test, *p* = 0.8112, HR = 1.109, 95% CI = 0.4275 to 2.204) within our cohort.

### 3.2 Relation of Clinical Parameters With Defined Histomorphological Biomarkers

To investigate a potential dependence of our histological features on already established clinical and pathological prognostic parameters, we assessed their respective associations individually in a subsequent analysis. Within our cohort, we did not find any significant associations between TB and PD‐L1 status (CPS: Mann–Whitney test, *p* = 0.6424; TPS: Mann–Whitney test, *p* = 0.4471), nor did we find any between PDC (CPS: Mann–Whitney test, *p* = 0.4147; TPS: Mann–Whitney test, *p* = 0.4853) or TSR (CPS: Mann–Whitney test, *p* = 0.7670; TPS: Mann–Whitney test, *p* = 0.3368) and immunohistochemical PD‐L1 expression. Conversely, a high level of sTILs infiltration was significantly associated with a high PD‐L1 expression level as assessed by CPS (Mann–Whitney test, *p* = 0.0158), whereas PD‐L1 expression measured by TPS (Mann–Whitney test, *p* = 0.6381) exhibited no significant correlation (see Figure S1).

We did not observe any statistically relevant associations of TB and PCD with T stage (TB: Kruskal Wallis test, *p* = 0.2619; PDC: Kruskal Wallis test, *p* = 0.2455) or primary tumor localization (TB: Kruskal Wallis test, *p* = 0.2331; PDC: Kruskal Wallis test, *p* = 0.4219) within our data set; see Figure S2.

**FIGURE 3:**
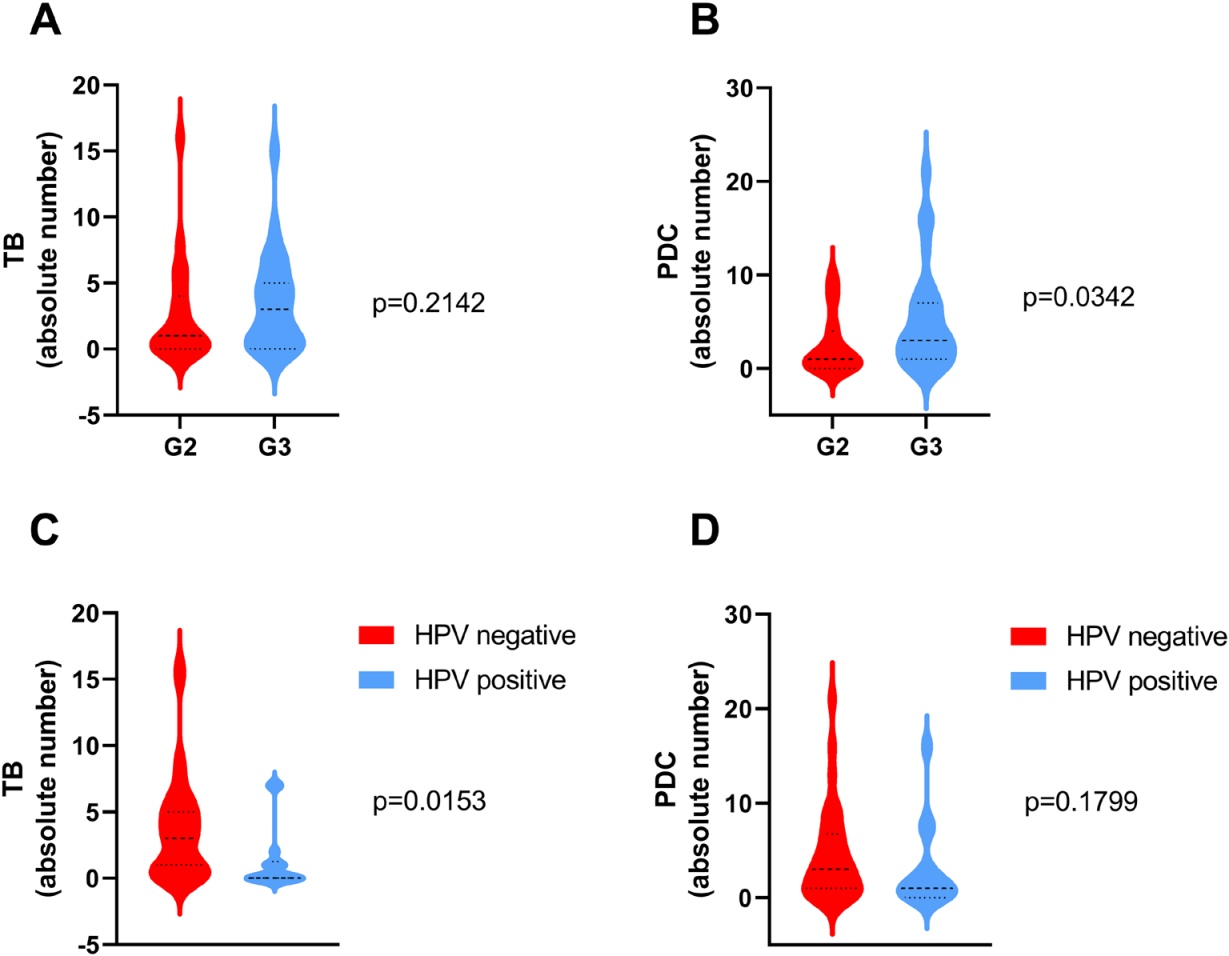
Correlation of TB and PDC with histopathological features within our cohort. Panels A and B illustrate the relationship of histopathological grading with TB and PDC, respectively. Panels C and D depict the associations of TB and PDC with HPV tumor status. In each panel (A–D), the median is indicated by a horizontal line, with the upper and lower bounds delineating the interquartile range.

Furthermore, only PDC was associated with the conventional histopathological grading (G1‐3) which by convention rates the overall degree of neoplastic cell differentiation (Mann–Whitney test, *p* = 0.0342), whereas TB formation was not associated (Mann–Whitney test, *p* = 0.2142). Interestingly, negative HPV tumor status was associated with the formation of tumor buds (Mann–Whitney test, *p* = 0.0153), while PDC (Mann–Whitney test, *p* = 0.1799) and sTILs (Mann–Whitney test, *p* = 0.7949) were not associated (see Figure S3 and Figure 3).

### 3.3 Proposal of a New Prognostic Scoring System

In order to establish a practical and simple scoring system based solely on H&E‐derived histomorphological features, we have assigned an individual point rank to all carcinomas according to the presence of each feature. Points are assigned for either TB or PDC, and a total score is calculated; see Table 2 for an overview of cutoffs per parameter. This final score can subsequently be used to rank the carcinomas within our proposed grading system as either grade I (2 points), grade II (3–4 points), or grade III (6 points) tumors.

**TABLE 2:** Composition of a novel grading system by the histopathological parameters TB and PDC.

Number of tumor buds (TB)	Number of poorly differentiated clusters (PDC)	Points
0–2	0–3	1
3–6	4–7	2
> 7	> 8	3

*Note:* Final grading after addition of TB‐points and PDC‐points: 2 points = grade I. 3–4 points = grade II. 5–6 points = grade III.

**FIGURE 4:**
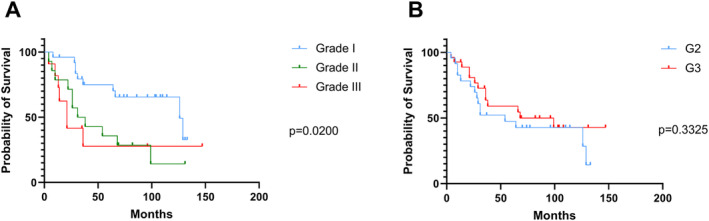
Prognostic relevance according to the proposed novel grading system (A) compared to the conventional grading system (B). Statistical analysis in both panels (A and B) was performed using the log‐rank test.

Within our patient cohort, 25 tumors were grouped grade I, 14 tumors were grouped grade II, and 11 tumors were identified as grade III. This scoring system effectively stratifies patients into 3 risk groups for OS (Log‐rank (Mantel‐Cox test), *p* = 0.0200; see Figure 4A). Hereby, our proposed system, which identifies both a distinct group with superior prognosis and patients at higher risk, surpasses not only single parameter‐based risk stratification models employing solely TB or PDC as single features (see Figure S4) but could serve even more useful in cases where risk assessment based on traditional grading parameters fails. This system outperformed traditional histologic grading, which was not associated with OS (Log‐rank (Mantel‐Cox test), *p* = 0.3325, HR = 1.450, 95% CI = 0.6840 to 3.073; see Figure 4B).

## 4 Discussion

Even though significant advances have been made in recent years, with the introduction of new immunotherapeutic options into the armamentarium available for the treatment of head and neck squamous cell carcinoma, the effectiveness of first‐ and second‐line immune checkpoint inhibition therapy remains modest [35]. This highlights the importance of biomarker development to sufficiently guide therapy assignment and prognostication [36]. Given the rapidly evolving field of immunotherapy and the escalating healthcare costs associated with increasingly expensive diagnostic procedures, there is value in widely available and globally accessible low‐cost approaches. That said, various histomorphological parameters, assessable directly on H&E slides, were analyzed in this study in 50 patients with high‐risk HNSCC (namely at least two lymph node metastases (LNM)). The selected tissue‐derived parameters, encompassing TB, PDC, sTILs, as well as the TSR, were subsequently correlated with OS and clinical characteristics, including HPV tumor status, primary tumor localization, and PD‐L1 expression. Hereby, no influence of primary tumor localization, PD‐L1 expression measured by CPS and TPS, T stage, or the conventional grading system was observed on TB and PDC. These two histomorphological predictors were subsequently successfully put to test as the foundation of a novel grading system designed to enhance prognostication.

### 4.1 Histomorphological Biomarkers in HNSCC

Within the last years, TB has been in focus of various studies as a fast and easy‐to‐employ histomorphological biomarker in HNSCC [37, 38]. However, given the established significance of biomarkers such as HPV tumor status and UICC stage, alongside the growing financial pressures on healthcare systems, our study diverges from previous research by concentrating on high‐risk HNSCC patients with the aim to evaluate whether TB could provide an enhanced prognostic assessment in this specific patient cohort. Comparable to our study, the teams of Stögbauer et al. and Channappa Niranjan et al. investigated the prognostic significance of TB in HNSCC tumors; despite differences in patient cohort composition compared to our study, both Stögbauer et al. and Channappa Niranjan et al. demonstrated a significantly poorer prognosis associated with high TB [37, 39]. These findings are further supported by a meta‐analysis conducted by França Vieira e Silva et al., encompassing 42 HNSCC studies, which confirmed decreased OS in cases of high TB formation [40]. A distinct difference between the aforementioned study by Channappa Niranjan et al. and our study presented here lies in the cutoff values chosen to define “TB positivity”—Channappa Niranjan et al. required five buds per high‐power field (HPF; 40×) whereas our scoring requires solely a minimum of 3 buds (×20) [39]. In contrast, Stögbauer et al. included a larger patient cohort (*n* = 331) when applying another different cutoff value to define “TB positivity” (Stögbauer et al. required six buds per digital high‐power field (97.464 μm^2^)). Hereby, the authors did not only distinguish between TB‐negative and ‐positive cases but further introduced a multi‐tiered classification system ranging from absent (0 buds) to weak (1–5 buds), moderate (6–14 buds), and strong (≥ 15 buds) [37].

The current lack of consensus on both the optimal cutoff value and the employed lens objective for tumor bud counting necessitates further studies to establish standardized cutoff values enabling a refined and reproducible stratification system. When selecting the parameters for this study, careful attention was paid to using parameters/cutoff values that have already been implemented in previous study designs exploring TB in HNSCC. Therefore, our choices are supported by existing literature, as summarized by a recent review by Togni et al. [30].

Among the histomorphological features analyzed in our study, PDC were shown to significantly correlate with decreased OS when present in high numbers. To our knowledge, PDC have not been explored within the context of HNSCC in existing literature to such extend. However, Miyazaki et al. have demonstrated the prognostic relevance of PDC in carcinomas of the external auditory canal, where a high prevalence of PDC correlated with reduced OS [18]. While representative studies on PDC in HNSCC are lacking, the prognostic value of PDC is well established in other malignancies, such as colorectal cancer [41, 42]. Further investigation into PDC in HNSCC is warranted to validate the prognostic significance of our promising finding also in other HNSCC cohorts/subgroups and potentially facilitate the integration of PDC analysis into clinical practice.

Contrary to TB and PDC, the assessment of sTILs did not demonstrate a significant impact on patient outcomes in our study. This finding diverges from existing literature, where TILs have been consistently established as independent prognostic biomarkers in numerous studies [43, 44, 45]. Using tissue microarrays, Spector et al. postulated that the extent of CD4/CD8/ FoxP3 positive TILs was associated with improved survival in a cohort of 464 HNSCC patients [43]. Potential explanations for this discrepancy could stem from methodological differences in TILs analysis: while our study evaluated sTILs on H&E slides, previous studies of not only our group but also, for example, Spector et al. utilized immunohistochemical methods for TIL assessment [43, 45]. Additional factors, such as our distinct focus on stromal infiltrating lymphocytes as well as the selective composition of our cohort, which included solely patients with locally advanced HNSCC and at least two LNM, may also contribute to the reported differences.

With regard to Figure S2, our data clearly display independence of both TB and PDC with regard to the respective primary tumor localization but also T stage, making it suitable for use as a potential biomarker in HNSCC across all stages and various subsites. As a limiting circumstance, it should be noted that the subgroups studied in our particular high‐risk cohort could not be balanced a priori due to the small sample size meeting our inclusion criteria, which naturally limits their statistical power and thus potentially their generalizability to cohorts with different characteristics.

### 4.2 The Association of Histological Markers and HPV/PDL‐1 Status

The presented findings of our study regarding higher TB in HPV‐negative cases are in line with previous findings from Stögbauer et al. [37]. Potential explanations for such an increased incidence of TB in HPV‐negative cases may arise from distinct tumor microenvironmental characteristics and divergent immune responses between HPV‐negative and HPV‐positive tumors [46]. Additionally, observed differences in TB patterns may be attributable to distinct variations in the epithelial‐to‐mesenchymal transition (EMT) processes between HPV‐positive and HPV‐negative tumors [47].

With the expanding role of immune checkpoint inhibitors (ICI) in the therapeutic management of HNSCC, the search for factors influencing therapeutic outcomes and diagnostic parameters is ongoing [48]. To enhance the efficacy of ICI, it is critical to analyze potential influencing factors. Therefore, we examined the correlations between histomorphological features—such as TB, PDC, sTILs, and TSR—and PD‐L1 expression, as defined by CPS and TPS. To date, studies exploring these correlations in HNSCC are lacking. However, in pancreatic and colorectal adenocarcinomas, few studies have reported higher TB in PD‐L1‐positive cases [49, 50]. Such contrasting results between reported findings in other cancer types and our findings presented here emphasize the necessity for further studies in HNSCC to establish robust conclusions regarding the relationship between PD‐L1 expression and TB.

### 4.3 Contribution of Histological Biomarkers to Novel Risk Models

Tumor prognostication has traditionally relied on histomorphological grading, which is commonly based on the degree of differentiation of neoplastic cells or the phenotypical aspects of neoplastic lesions at the invasive tumor border. However, the non‐significant differences in OS between different tumor grades (in our study: based on the overall degree of neoplastic cell differentiation) within our high‐risk patient cohort highlight the urgent need for novel strategies to enhance prognostic accuracy, especially within the context of ongoing debates regarding the questionable benefits of tumor‐agnostic grading systems across various tumor entities of squamous cell carcinomas. Previous studies have proposed novel risk models that include not only TB as a single parameter but its combination with, for example, depth of infiltration (Almangush et al.), cell nest size (Boxberg et al.), traditional WHO grading (Elseragay et al.), or the Glasgow environment score (GM score: based on the tumor‐stroma percentage and the inflammatory status [51, 52, 53]; Yu et al.) [54, 55, 56, 57, 58].

In our study, we propose a cost‐effective and straightforward to implement novel grading system for high‐risk HNSCC patients based on TB and PDC, enabling prognostication solely by means of H&E staining. In line with our findings, Stögbauer et al. assessed the prognostication of HNSCC tumors using another novel cellular dissociation grading (CDG) system, which incorporates TB and minimal cell nest size (MCNS). Their three‐tier system demonstrated improved OS, particularly in subgroup analyses of HPV‐positive tumors [37]. Although both Stögbauer et al.'s study and ours yielded similar outcomes, it is important to note that the TCGA (The Cancer Genome Atlas) cohort used by Stögbauer et al. included tumors across low, intermediate, and high‐risk categories, whereas our study focused exclusively on enhancing prognostication within a challenging cohort of high‐risk tumors. Nonetheless, both studies demonstrated that the respective grading systems each outperform the conventional grading system. To facilitate the integration of these systems into routine clinical practice, further research is required, including prospective studies with larger patient cohorts and more comprehensive analyses of HNSCC subgroups.

### 4.4 Limitations

From a critical perspective, one has to consider the sample size of 50 patients in our study. Given our objective to improve prognostication within a particularly challenging cohort of high‐risk tumors, we prioritized histomorphological analyses on fully resected specimens. This approach, however, is less common in advanced tumor stages, where complete resection is often not feasible. While an implementation of additional patients, for example, with alternating tumor stages/tumor biology would result in a greater patient cohort, it would also increase biological and clinical variability and therefore potentially hamper the uniformity of our cohort of interest. Secondly, all patients included in our study cohort received different treatment regimens, with some receiving surgery alone and others receiving a combination of surgery with radiotherapy and, where appropriate, chemotherapy/epidermal growth factor receptor (EGFR) inhibition (*cetuximab*). According to good clinical practice, the individual treatment choice is based not only on clinicopathological factors such as UICC stage but also on patient‐specific factors (age, comorbidities), both contributing per se to potentially varying outcomes. Though this variability reflects clinical routine in oncology, the particular difference in treatment modalities introduces potential confounding factors during statistical data analysis, which may not only affect the internal validity of the results presented but also limit the applicability and generalizability to broader patient populations. Future studies could therefore incorporate additional methodological strategies such as rigorous patient stratification (employing e.g., propensity score matching or even randomization techniques whenever feasible) to externally revalidate our conclusions. Last but not least, it is essential to establish standardized cutoff values and assessment protocols when integrating a novel grading system or risk model comprising, for example, TB and PDC into routine clinical practice—however, such standards are currently lacking in HNSCC.

## 5 Conclusion

Taken together, our study proved the prognostic value of TB and determined the prognostic implications of PDC in high‐risk HNSCC as an independent prognostic biomarker and a possible alternative to clinically established biomarkers. The cost‐effectiveness and broad availability of these methods support their potential integration into routine clinical practice. The implication of a novel proposed grading system showed superior prognostication in comparison to conventional grading approaches; however, future studies need to validate our findings.

## Author Contributions

M.L., S.K., F.B., S.W., J.P.K., L.B., and B.S. contributed to the conception and design of the study. S.S. provided HPV data. G.G.K. and M.W. provided and analyzed tissue samples. M.E. performed the immunohistochemical staining. M.K. and G.G.K. analyzed the database and provided statistical analyses. M.K. and G.G.K. wrote the first draft of the manuscript. M.L. and L.G.T.M. wrote sections of the manuscript. All authors contributed to the article and approved the submitted version.

## Conflicts of Interest

The authors declare no conflicts of interest.

## Supporting information


Figure S1

## Data Availability

The datasets generated during and/or analyzed during the current study are available from the corresponding author on reasonable request.
