# Supplementary material for: Optimizing Prognostic Assessment in High‐Risk Head and Neck Squamous Cell Carcinomas: The Impact of Tumor Budding and a Novel Histomorphological Scoring System
Source: Cancer Med. 2025 Feb 15;14(4):e70685. doi: 10.1002/cam4.70685 (PMC11829162; doi:10.1002/cam4.70685)
Supplement: Supplementary file 1 — Figure S1 [file CAM4-14-e70685-s001.docx]

Supplemental Information: **Manuscript ID:** CAM4-2024-10-6047

Title: Prognostic Implications of a Proposed Histomorphological Risk Factor based Scoring System in Head and Neck Squamous Cell Carcinomas with Positive Lymph Node Status


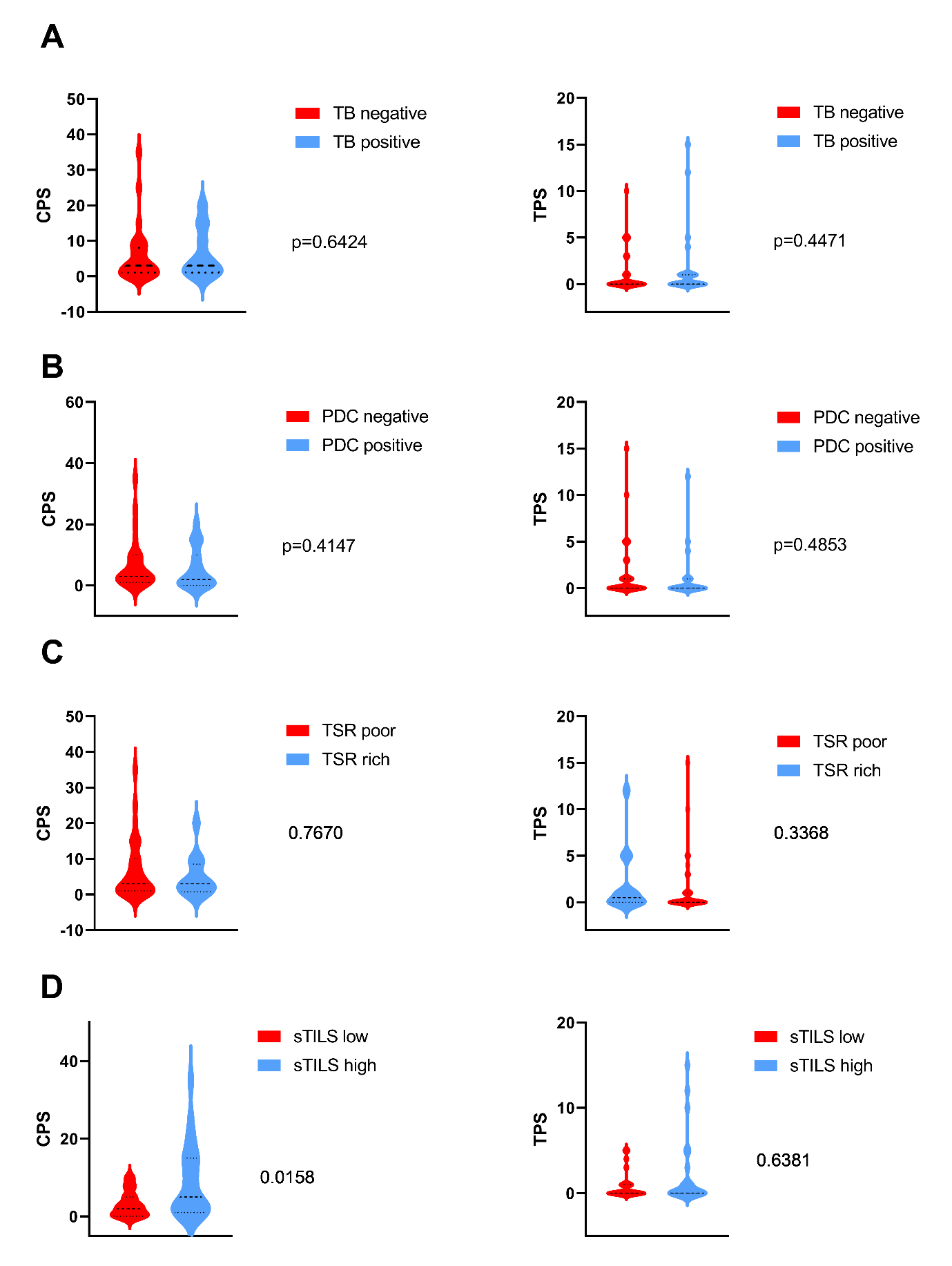


**Supp. Figure 1.** Correlation of histomorphological features including (A) TB, (B) PDC, (C) TSR and (D) sTILs with PD-L1 expression assessed by CPS and TPS. In each panel (**A-D**), the median is indicated by a horizontal line, with the upper and lower bounds delineating the interquartile range.

**
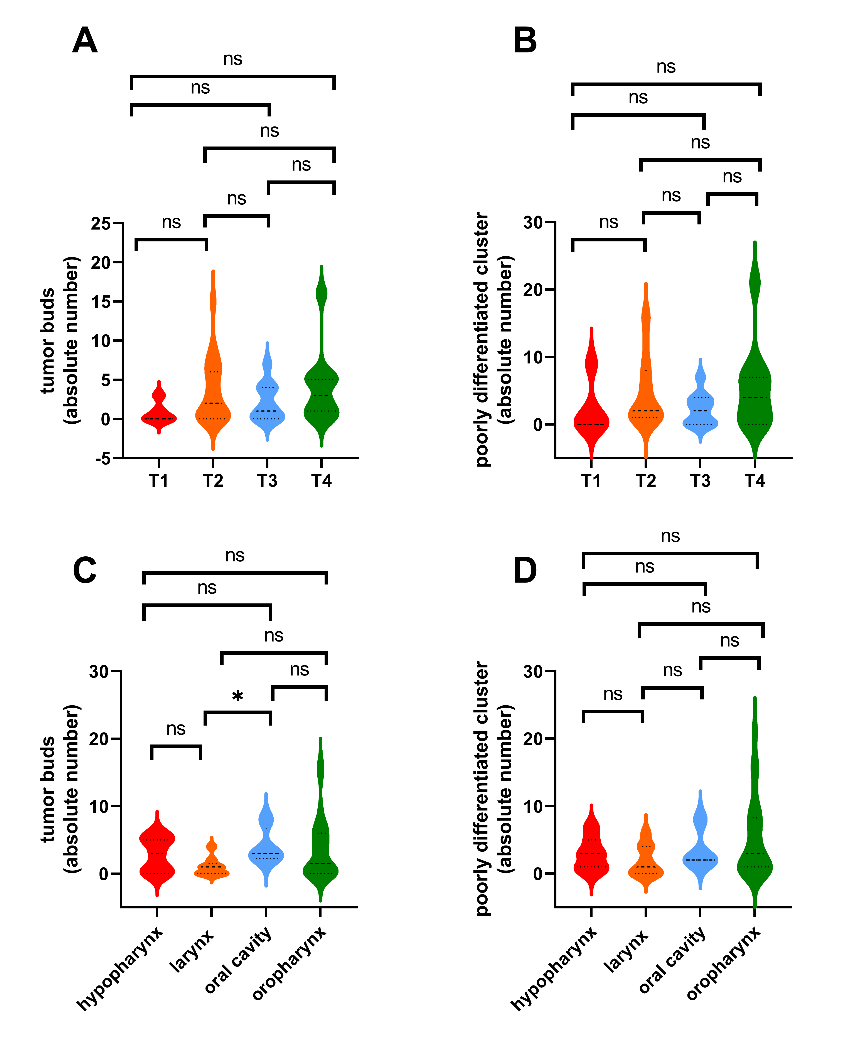
**

**Supp. Figure 2.** Association of T-stage with (A) absolute number of tumor buds and (B) absolute number of PDC. As well as association of primary tumor localization with (C) absolute number of tumor buds and (D) absolute number of PDC. In each panel (A to D), the median is indicated by a horizontal line.


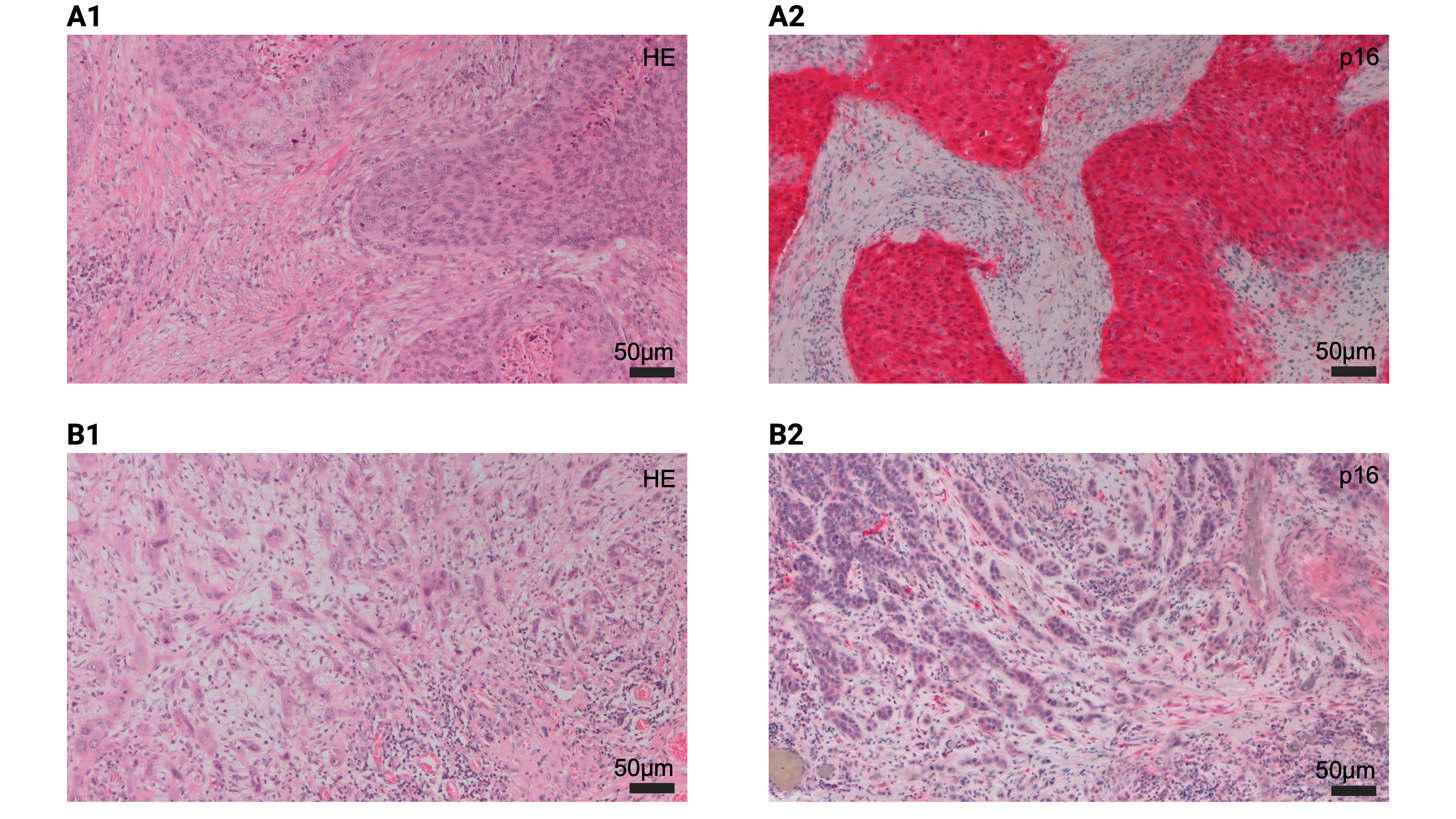


**Supp. Figure 3.** Visualization of HPV association determined by p16 immunohistochemistry and corresponding hematoxylin and eosin (HE) stained section of the same tumor/patient. Patient A shows a pushing infiltration zone without bud formation (**A1**) and a classical block-type p16 immunohistochemistry (**A2**). Patient B shows high TB formation (**B1**) and no relevant p16 positivity – as in diagnostic routine, single stained cells as well as faint cytoplasmatic staining without nuclear aspects are not considered (**B2**).


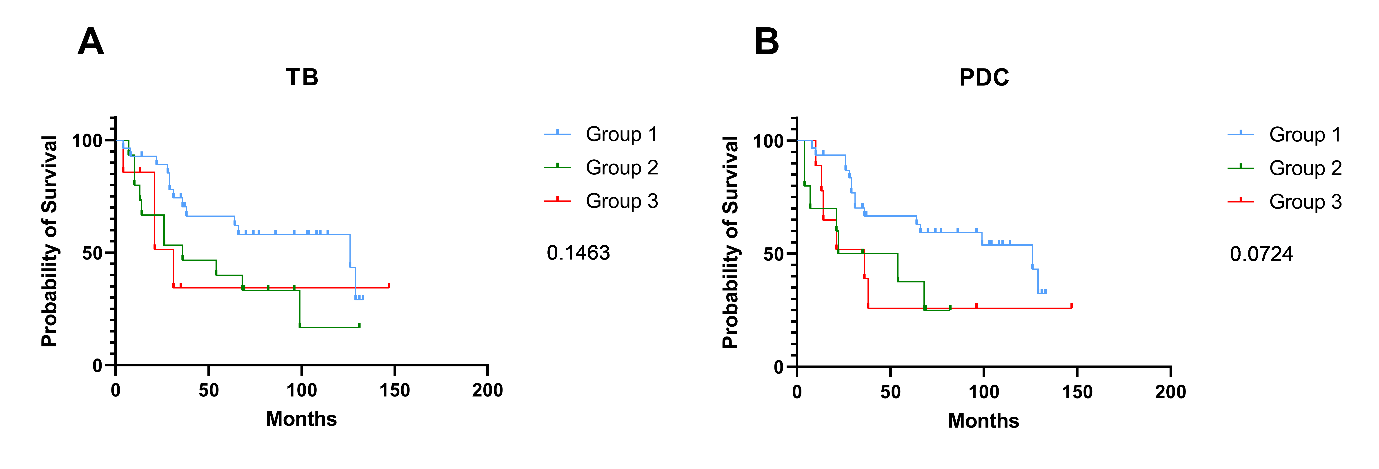


**Supp. Figure 4.** Prognostic significance of the subgroups (A) TB (number of buds: group 1 = 0-2, group 2 = 3-6, group 3 = >7) and (B) PDC (number of clusters: group 1 = 0-3, group 2 = 4-7, group 3 = >8) which contribute to the final grade of the novel grading system. Statistical analysis in both panels (A) and (B) was conducted using the log-rank test.
